# Lipidomic Comparison of Detergent‐Soluble and Detergent‐Resistant Lipid Domains Isolated From Rat Cerebellum and the Effect of Hypoxia–Ischemia Using UPLC‐HDMS^E^

**DOI:** 10.1002/jms.70000

**Published:** 2026-01-05

**Authors:** Samuel A. Krug, Min He, Ningfeng Tang, Cynthia Bearer, Maureen A. Kane

**Affiliations:** ^1^ Department of Pharmaceutical Sciences University of Maryland School of Pharmacy Baltimore Maryland USA; ^2^ Department of Pediatrics University of Maryland School of Medicine Baltimore Maryland USA

**Keywords:** hypoxic ischemia, lipid membrane, lipidomics, liquid chromatography–tandem mass spectrometry, membrane domains

## Abstract

Hypoxic ischemic encephalopathy (HIE) due to insufficient oxygen or blood flow to the brain can result in inflammation, impaired neurodevelopment, or death. Changes to microdomain lipid composition may contribute to altered structure and function of key regulatory processes; however, knowledge regarding changes to membrane lipid composition after HIE is incomplete. Here, we describe the application of untargeted lipidomics to investigate the impact of hypoxia–ischemia on detergent‐resistant membrane (DRM) and detergent‐soluble membrane (DSM) domains fractionated from the cerebellum in a rat model of HIE. Lipidomics utilized ultra performance liquid chromatography coupled to data independent tandem mass spectrometry with traveling wave ion mobility. Lipid alterations specific to the DRM domains after hypoxia–ischemia included lysophospholipids, sphingomyelins, ceramides, triglycerides, and phosphatidylethanolamines, which are lipid species that have been linked to cognitive and neuronal impairment. The advances in lipidomics have enabled the tools available to study lipid composition. These data may provide insight into membrane disruption after HIE in relation to lipid composition and concentration.

## 1 Introduction

Hypoxic–ischemic encephalopathy (HIE) due to insufficient oxygen or blood flow to the brain can result in inflammation, impaired neurodevelopment, or death [1]. HIE is a perinatal insult that changes the protein distribution in organized lipid microdomains in the developing cerebellum [2]. One such protein, L1, is a cell adhesion protein of the immunoglobulin superfamily associated with lipid rafts in the cerebellum during specific phases of brain development [3, 4]. L1 mediates neurite elongation and migration of neuronal precursors; this function is dependent on its association with lipid raft domains [5, 6, 7, 8]. A variety of perinatal insults can change the L1 distribution to lipid rafts disrupting cerebellar development including HIE, ethanol exposure, toluene exposure, and bilirubin excess [1, 4, 9, 10]. Molecules like choline, crucial for structural components of cells, have been shown to mitigate ethanol‐induced disruption of lipid rafts [8]. Choline‐containing phospholipids are a component of lipid rafts in cell membranes, including phosphatidylcholine and sphingomyelin. As such, changes to the composition of ordered lipid domains may be central to the disruption of lipid raft–mediated signaling in the developing cerebellum.

Lipid raft formation is driven by lipid–lipid interactions that influence the size and lipid composition of the domain [9]. The microdomains within membranes can be isolated into two distinct fractions. Ordered lipid domains are detergent‐resistant, and within this manuscript are termed detergent‐resistant membranes (DRM). The other portion of lipid domains that can be solubilized by detergent are termed detergent‐soluble membranes (DSMs). While there are some imperfections in isolation techniques, detergent‐soluble and detergent‐insoluble isolates are still routinely used for studying lipid microdomains in order to enrich fractions within the membrane [11]. DSM and DRM are distinguished by the extent of flexibility and permeability, which is much lower for DRM domains [12, 13]. DRM domains are considered to be rich in saturated lipids, sterols, sphingolipids, and triglycerides (TGs) and are more tightly packed. Many lipids do not exclusively exist in one domain or the other [14, 15]. Fractionation methods indiscriminately enrich lipid domains from both the plasma membrane and intracellular membranes. This can be mitigated by further subcellular fractionating organelles before analysis, but for this study, the data reported are for DRM and DSM in the entire cell.

Lipid domains may differ according to their cellular location, for example, between the inner and outer leaflet [16, 17, 18]. Phosphatidylethanolamines (PEs) are preferentially localized on the inner leaflet of the plasma membrane. Intracellular membranes, like the mitochondria or Golgi apparatus, have been shown to have different lipid compositions in the DRM domain. This has led to several hypotheses about the importance of lipid domains for transport and organization [19, 20, 21, 22]. Subdomains of the endoplasmic reticulum that interact with mitochondria, termed mitochondria‐associated membranes (MAMs), play roles in autophagy, signal transduction, and gene transcription—all with various lipid compositions.

Mass spectrometry–based approaches, particularly untargeted lipidomics, have allowed for more in‐depth study of lipid microdomains [23, 24, 25]. There are a number of studies that utilize lipidomics in order to qualitatively assess the changes during neurological diseases [19, 26, 27]. Fatty acid profiles were measured in blood samples taken from newborns with HIE and showed a reduction in omega‐3 PUFAs such as docosahexaenoic acid (DHA) and eicosapentaenoic acid (EPA). These results trended with poor neurological outcomes [28].

A study using mass spectrometry imaging observed lipid changes in murine brain and highlighted that severely damaged regions had an increase of *N*‐acylphosphatidylethanolamine and monosialogangliosides GM2 and GM3 [29]. Combined, these datasets inform on lipid changes after HIE but have limitations. For example, lipid compositions in the blood are not solely from the brain tissue but also are impacted by damage to other tissues, such as the heart.

This study was designed to study lipidomic changes in the DRM and DSM after HIE using ultra performance liquid chromatography coupled to data independent tandem mass spectrometry with traveling wave ion mobility as we have previously described [30]. This study used a rat pup model of neonatal HIE, which has been characterized previously for its impact on neuronal development [30, 31], specifically, the cell growth, proliferation, and refining of neuronal circuits that are critical in the first few weeks after birth [32]. Lipid domains are believed to facilitate cell signaling and protein trafficking, which are particularly important for initial stages of neurodevelopment in new born infants. By focusing analysis on cerebellar tissue, it is possible to understand lipidomic changes specific to the brain that can be used for future HIE injury intervention strategies [31]. Herein, we will describe the lipidomic analysis of both DRM and DSM samples isolated from the cerebellum of postnatal day 10 rats that have undergone HIE.

## 2 Materials and Methods

### 2.1 Hypoxic–Ischemic Injury Model

All animal procedures were reviewed and approved by the University of Maryland Baltimore Institutional Animal Care and Use Committee and adhered to the National Institutes of Health guide for the care and use of laboratory animals [31]. Hypoxic ischemia was induced in postnatal day 10 (PN10) rat pups as previously described. In brief, 10‐day‐old pups were first anesthetized with isoflurane. The right carotid artery was isolated, ligated, and severed to prevent blood flow to the brain. The Sham pups also underwent a small incision and suture to mimic the surgical condition. In order to mimic hypoxic conditions, pups (1–2) were placed in a jar continually flushed with 92% nitrogen/8% oxygen for 1 h. Sham pups were maintained under normoxia conditions during this incubation. Following hypoxic conditions, pups were placed in a large jar (ambient oxygen conditions) under hypothermia at 32°C for 4 h followed by normothermia for 1 h. Sham pups were maintained under normothermia conditions during this period. Tissue was then immediately collected for isolation. The final number of pups in each condition was Sham—*n* = 6 (three male, three female) and HI—*n* = 6 (three male, three female).

### 2.2 Isolation of DSM and DRM Fractions

Pups were decapitated, and the cerebellum was isolated as previously described [31]. Tissue was homogenized in Tris‐buffered saline containing 0.5% Triton X‐100 and supplemented with 10 μM sodium vanadate, 2 μM aprotinin, 100 pM cypermethrin, and phosphate inhibitor cocktail 1 and 2. Homogenates were incubated on ice for 30 min and then centrifuged at 13000 × g for 10 min at 4°C. From the resulting supernatant, 1 mL was mixed with 1 mL 80% sucrose and a discontinuous 5%–35%–40% sucrose gradient was formed by adding 4 mL 35% sucrose and 4 mL 5% sucrose on top of the initial homogenate solution. The samples were centrifuged at 180000 g for 24 h at 4°C using a Beckman SW41 rotor. One‐milliliter fractions were taken starting from the top of each gradient. Fractions were immunoblotted for GM1 ganglioside, and all fractions containing GM1 were pooled as the DRM fraction. All remaining fractions were combined as the DSM fraction.

### 2.3 Total Lipid Extraction

From the resulting combined fractions, 50 μL sample volume was combined with 10 μL of EquiSPLASH Lipidomix (Avanti Polar Lipids, Birmingham, AL) to serve as an internal standard. To create a “pooled QC” for quality control, 5 μL of each sample was added to the same tube. In order to extract lipids, 200 μL of 1‐butanol/methanol (1:1/v:v) with 5 mM ammonium formate was added to each tube, and then, samples were briefly vortexed and allowed to sonicate in ice for 1 h. After sonication, samples were then centrifuged at 13 000 g for 10 min and the supernatant was transferred to autosampler vials for analysis.

### 2.4 Untargeted UPLC‐HDMS^E^ Analysis

Untargeted lipidomic analysis was performed as previously described for detection and structural characterization of lipids in lysosomes isolated from cortex [30]. UPLC separation was performed using Waters ACQUITY system with a C18 CSH (2.1 × 100 mm, 1.7 μm) column (Waters, Milford, MA). Mobile Phase A was 10 mM ammonium formate in water/acetonitrile (40:60, v/v) with 0.1% formic acid, and Mobile Phase B was 10 mM ammonium formate in acetonitrile/isopropanol (10:90, v:v) with 0.1% formic acid.

HDMS^E^ analysis was carried out using a traveling wave ion‐mobility enabled hybrid quadrupole orthogonal acceleration time‐of‐flight mass spectrometer (SYNAPT G2‐S, Waters). Raw data were obtained from 100 to 1800 m/z for both positive and negative ionization modes. The first scan was set at low collision energy and used to determine the precursor ion, while the second scan ramped from 30 to 55 eV (high collision energy) in order to determine product ions. Leucine enkephalin (0.1 mg/mL in water: ACN/1:1/v:v with 0.1% formic acid) was used as a lock‐mass to validate high mass accuracy data and infused at a flow rate of 7.5 μL per minute. Ion mobility drift times were determined from the precursor ions using nitrogen as a drift gas with a wave height of 40 V and a velocity of 600 m/s.

### 2.5 Data Analysis and Feature Identification

Data were acquired using MassLynx v4.1 (Waters). Raw data in both ESI− and ESI+ mode were imported directly to Progenesis (Nonlinear Dynamics, Waters, v3.0). Initially, over 10 000 features were detected in both negative and positive modes combined. Peak integrity, abundance, *p*‐value (*p* < 0.05), and fold change (FC > 1.5) were used to initially filter peaks of interest. EquiSplash was included as an internal standard in each sample and used as a retention time marker for lipids from multiple classes. LipidMAPS (± 10 ppm), NIST MS/MS Library (± 10 ppm), and Metabolic Profiling CCS Library (± 10 ppm) were all utilized to putatively identify features according to mass error, fragmentation, and retention time compared with the known lipid class standards. The lipid class standards are provided in Figure S1 and Table S1. Peaks that were able to be identified were not confirmed by authentic analytical standards; therefore, the exact composition of acyl chains and degrees of unsaturation are predictions. As such, identifications like PE(P‐18:1/22:5) should be regarded as PE(P‐40:6) where 40 is the total number of carbons (carbon number) in the acyl *sn*‐1 and *sn*‐2 positions and 6 is the combined degrees of unsaturation. Pooled QCs (*n* = 4) were placed at the beginning, middle, and end of the run. Batches evaluated for QC performance by retention time shift within ± 0.2 min from the beginning to the end of the run and features putatively identified, including internal standards, had an abundance %CV < 15%.

### 2.6 Single‐Point Quantification of Lipid Species

EquiSplash was added at a known concentration of 10 μg/mL to each sample. A single‐point calibration was used to provide an estimated concentration for the putatively identified species in the sample. The area ratio for the peak area of the EquiSplash lipid was divided by the peak area of the identified lipid from that same lipid class and multiplied by the EquiSplash concentration in order to yield the concentration of the identified lipid in the sample in microgram per milliliter. It is important to note the sample concentration is that of a subcellular fraction and those DRM and DSM fractions encompass both the cell and organelle membranes from cerebellar tissue.

### 2.7 Statistical Analysis

Statistical analysis was performed on peaks using MetaboAnalyst v.6.0 and GraphPad Prism (v 10.4.2, La Jolla, CA). Multiple *t*‐test comparisons were performed with a false discovery rate of 1% using a two‐stage step‐up method of Benjamini, Krieger, and Yekutieli, and the *q*‐value was reported. Data generated in Progenesis were imported into MetaboAnalyst in order to construct PCA and heatmap plots. Significant peaks were normalized by using Pareto Scaling (mean divided by the standard deviation for each variable).

## 3 Results and Discussion

### 3.1 Putative Identification and Multivariate Analysis

**FIGURE 1:**
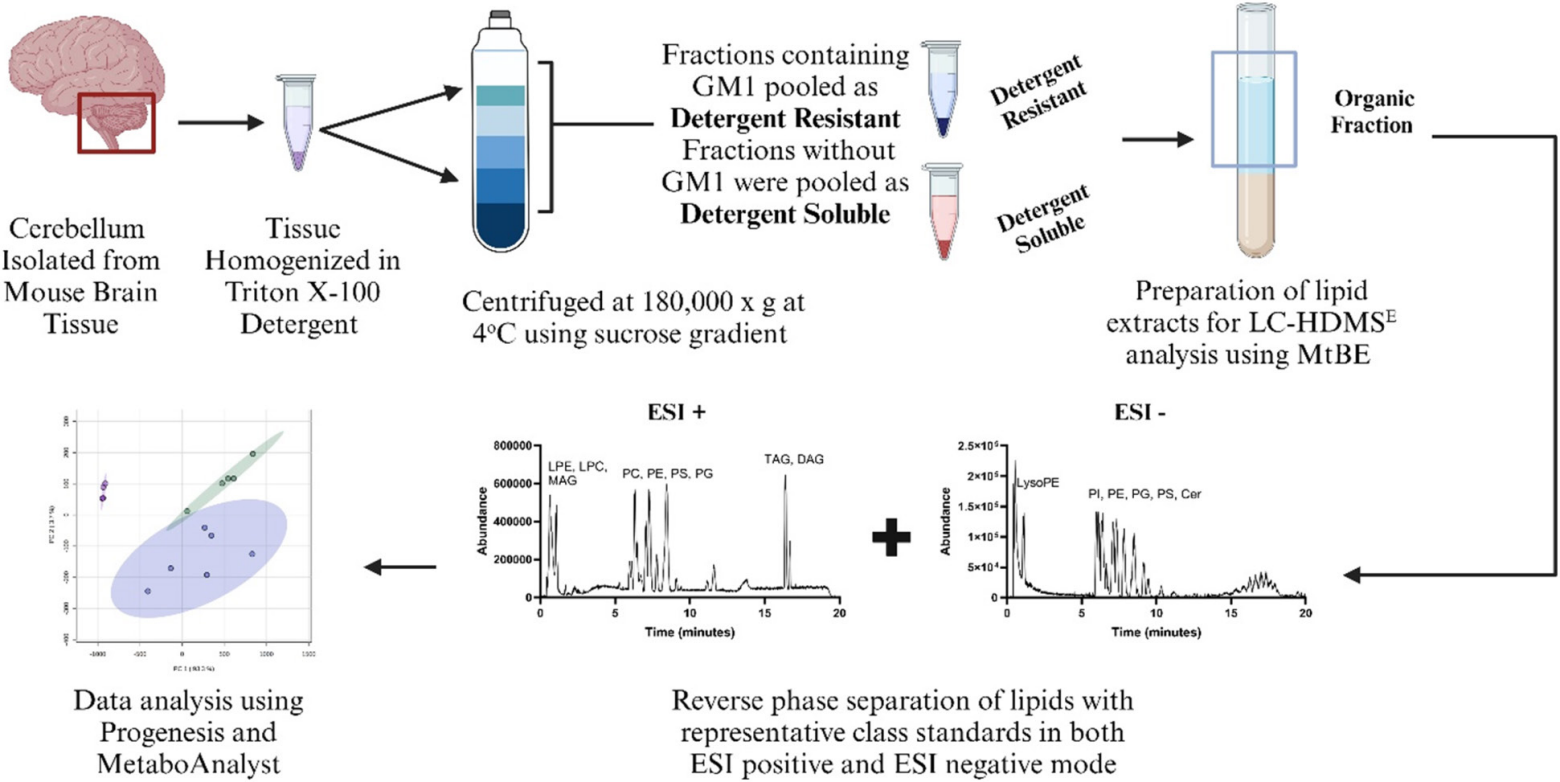
Workflow schematic for untargeted lipid analysis by UPLC‐HDMS^E^. Workflow scheme including a representative chromatogram of an extracted EquiSPLASH standard in ESI+ mode notating the typical elution regions for various lipid classes.

A workflow summary is provided as Figure 1. All putatively identified peaks and features are provided in Table S2, which details the retention time, experimental exact mass (*m/z*), ionization mode, drift time, mass spectrum peaks, and databases used to identify the analyte. Sixty‐seven features had significant changes (34 from ESI+ and 33 from ESI−), and 59 of these features were able to be identified by searching known databases; eight features were unable to be identified. The breakdown of the classes of lipids able to be identified was PE (28), phosphatidylserine (PS; 9), phosphatidylcholine (PC; 7), lysophosphatidylcholine (LPC; 5), lysophosphatidylethanolamine (LPE; 4), TG (3), phosphatidylinositol (PI; 1), and sphingolipid (SP; 2). Ten putatively identified lipids showed they were more abundant in the DRM domain (PE—4, TG—3, PC—2, SP—2). Tables S3 and S4 show the analytes with their designated lipid class as well as the concentration ± SEM.

**FIGURE 2:**
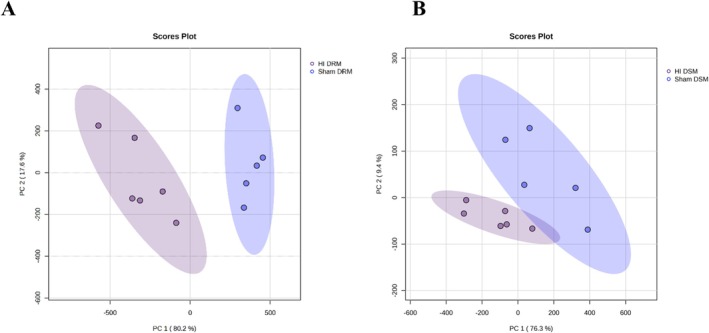
Lipid profiles in DRM and DSM are altered by HIE. (A) PCA plot comparing Sham (blue) and HI (purple) DRM fractions. PCA displayed a total of 97.8% of variance attributed to PC1 (80.2%) and PC2 (17.6%) components. Elliptical patterns show the 95% confidence interval for each group. (B) PCA plot comparing Sham (blue) and HI (purple) DSM fractions. PCA displayed a total of 85.7% of variance attributed to PC1 (76.3%) and PC2 (9.4%) components. Elliptical patterns show the 95% confidence interval for each group.

**FIGURE 3:**
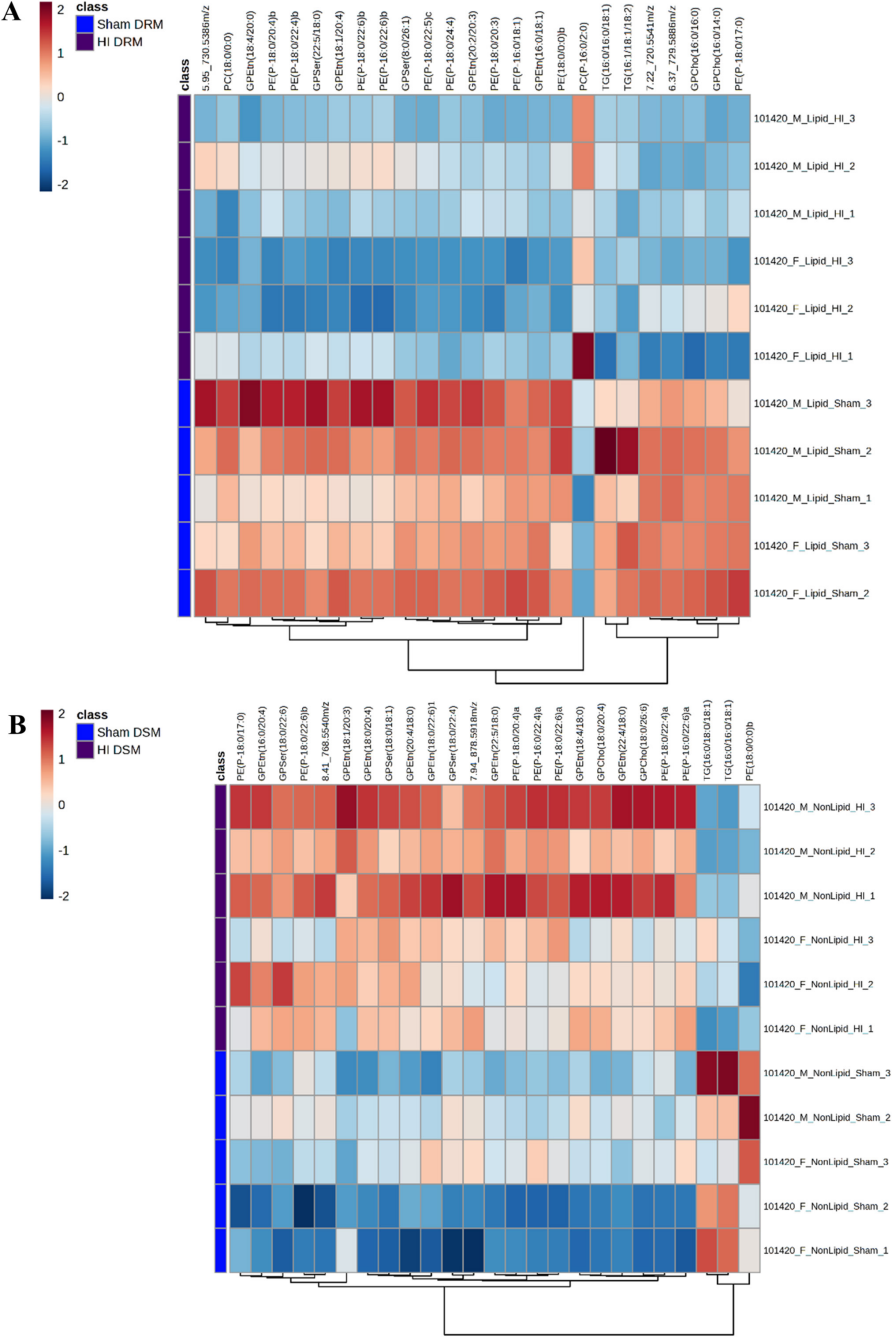
Lipid changes in DRM and DSM after HI. Hierarchical (Euclidean Ward) clustering of Sham (blue) and HI (purple) in (A) DRM fractions and (B) DSM fractions using the top 25 features ranked by their *p*‐value analyzed in MetaboAnalyst 6.0.

The PCA plots for the comparison of the lipid profile of DRM and DSM samples between the Sham and HIE conditions are shown as Figure 2A,B respectively. An example of PCA including QC samples has been provided as Figure S2. Heatmaps were constructed comparing the lipid abundance in the DRM and DSM fractions (Figure 3A,B) between individual samples of the Sham and HIE conditions for the top 25 features ranked by their *p*‐value. Heatmaps for all analytes detected are shown in Figure S3 for DRM and Figure S4 for DSM.

### 3.2 Experimental Approaches to Lipid Membrane Analysis

Previous approaches to studying membrane domains include microscopy and molecular simulation to capture membrane interactions. Due to the dynamic nature of membrane domains, each technique is limited in what information is provided. Our approach for studying these domains in order to add to current literature was to use an in vivo isolation in order to appropriately capture all of the lipid classes and species that are endogenously abundant. Fluorescent probes have been used experimentally to visualize the different kinetic behavior in DRM and DSM fractions, which has allowed researchers to understand the timeframe and scale at which microdomains form [33, 34, 35, 36]. Proteomic analysis has been utilized to understand lipid raft–associated proteins and investigate the influence of membrane structure on cell signaling. Initially, lipid raft isolation techniques would use antibodies to determine the lipid raft domain based on the prevalence of lipid raft–associated proteins. The caveat with this type of analysis is that proteins with a large number of transmembrane domains, such as caveolin, will create an inherent structure, such that the number of transmembrane domains needs to be considered when designating proteins for DRM and DSM domain markers. Concentration of detergent, ratio of detergent to tissue, and temperature during isolation have all been shown to affect the resulting fractions; however, detergent‐based isolation techniques yield reproducible results compared with other isolation methods [10, 37, 38]. Characterizing impacts to the metabolome and resulting phenotype of perturbing membrane lipid domains is still an important unanswered research question and may lead to future therapeutic targets.

### 3.3 Lysophospholipids Are Altered in the DRM Domain as a Result of HI Insult

**FIGURE 4:**
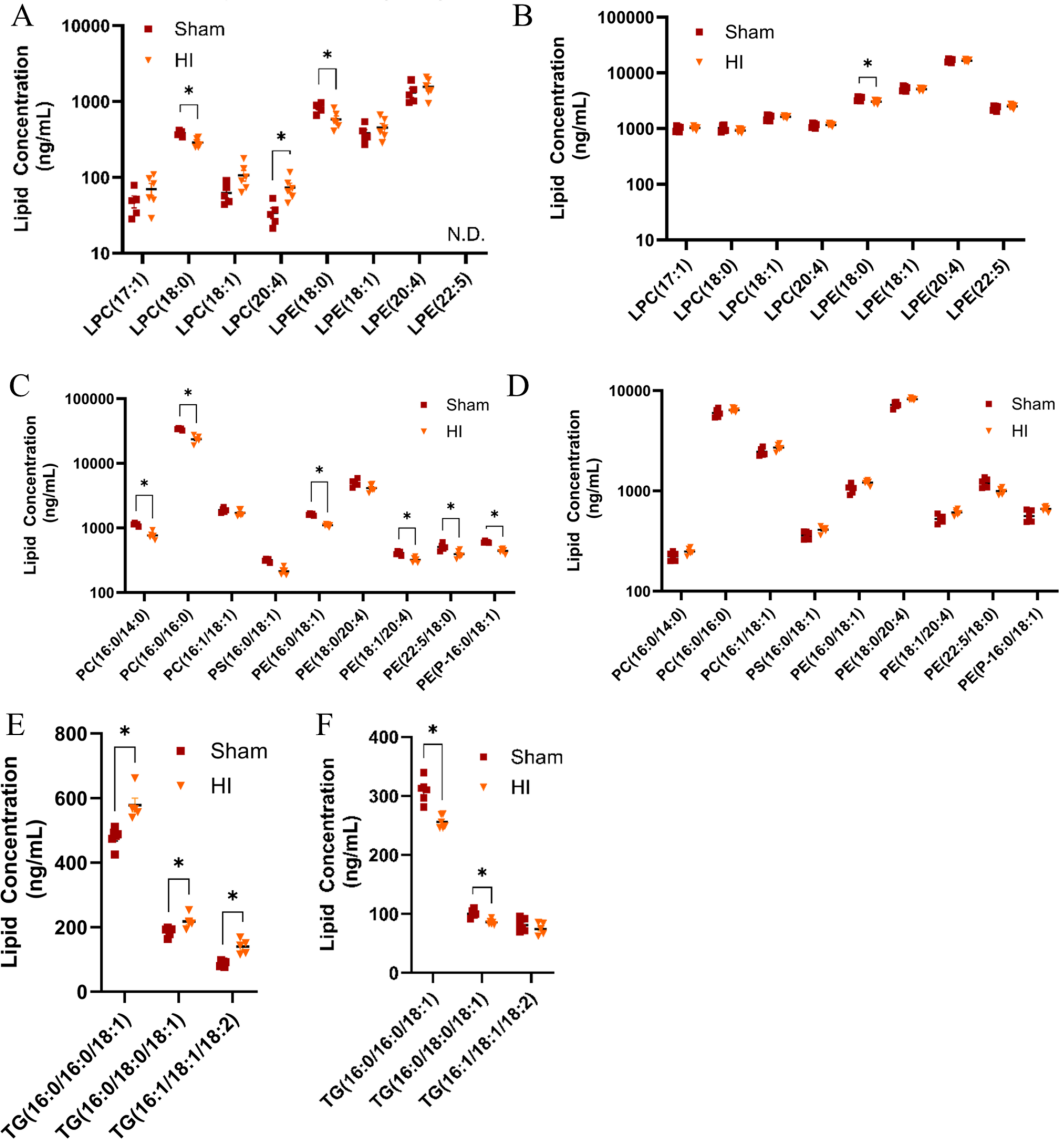
More changes in lipids after HI are observed in DRM than DSM. Comparison between Sham and HI DRM (A, C, E) and DSM (B, D, F) domains broken up by lipid classes. Panels A and B depict lysophospholipids, C and D show glycerophospholipids, and E and F show triglycerides. Note that some graphs have log_10_ *y*‐axis due to the large variation of compositional abundance in lipids present. * indicates *p* < 0.05 using Student's *T*‐test; N.D. indicates that analyte was not detectable in the sample.

Our results in Figure 4A,B compare the eight different lysophospholipids putatively identified during the study including four LPC and four LPE. The changes in their abundance in the DRM (Figure 4A) and DSM (Figure 4B) between the Sham and HIE conditions are noted. All of the saturated and unsaturated LPCs are unchanged in the DSM fraction. In the DRM, we observed a significant increase in LPC(20:4) in the HIE condition as compared with Sham. LPC (18:0) was the most abundant identified lysophospholipid in the DRM fraction and had a significant decrease that was not observed in the DSM fraction. For LPE, there is a decrease after HIE for LPE (18:0) in both the DSM and DRM samples, indicating that this decrease is not constrained to a specific membrane group. Lysophospholipids serve as important signaling molecules and help to traffic fatty acid chains to the brain [39]. DHA decreases in numerous neurological diseases such as Alzheimer's and schizophrenia and exhibits anti‐inflammatory and anti‐apoptotic properties. A number of studies have tried to supplement brain injury with DHA [39, 40]. The liver will synthesize PCs containing DHA from LPCs, resulting in a PC‐DHA complex necessary for transporter‐mediated interaction to facilitate DHA reaching the brain [41]. This is because transporters, like MFSD2A, will not transport free fatty acid chains into the cell for metabolic use and the LPCs will act as a delivery system [42].

Finally, we observed that unsaturated lysophospholipids were compositionally more abundant in the DSM compared with the DRM fraction. This is consistent with previous findings that DRM is typically composed of lipids with saturated acyl chains and the Van der Waals force helps to maintain the rigidity and structure of the domain [43]. Introducing degrees of unsaturation will allow the acyl chain to “kink” and disrupt these interactions, making the domain more fluid.

### 3.4 Glycerophospholipids Are Decreased in DRMs, but not in DSMs

Figure 4C,D show that almost all glycerophospholipids (PC, PS, PE) were decreased after HIE in the DRM, but this change was not observed for the same lipid species in the DSM. This is consistent with the expectation that HIE insult would disrupt the composition of DRMs. Our findings also align with altered lipid metabolism and transport. Above, we discussed how LPCs are important for transport of essential fatty acids to the brain. LPCs can be generated from the cleavage of acyl chains by phospholipases from existing PCs or can be a result of multistep enzymatic processes. Phospholipases, such as PLA2G4A/cPLA2 (phospholipase A2, group IVA [cytosolic, calcium‐dependent]), are activated in expression and activity in the brain during neurodegenerative diseases, after spinal cord injury, and after traumatic brain injury [27, 44, 45, 46, 47]. PLA2G4A/cPLA2 activation after controlled cortical impact in a model of traumatic brain injury saw reductions in similar PC and PE lipids after TBI injury as we reported here after HIE, including PC(16:0/14:0), PC(18:0/20:4), PE(18:0/20:4), PE(18:1/20:4), PE(22:5/18:0), and PE(P‐16:0/18:1) [47]. Damage to lysosomal membranes after controlled cortical impact led to impaired autophagy and neuronal cell death.

PS can be converted to PE by phosphatidylserine decarboxylase (PSD1), which primarily resides in the mitochondria and is responsible for maintain energy balance in the cell. PEs can further undergo conversion to PCs by PE *N*‐methyltransferase (PEMT); this process occurs in the endoplasmic reticulum. Together, this supports that membrane fractions we are observing are not solely from the plasma membrane but also from cell organelles as well. The class of lipids impacted the most are PEs, and this class of lipids is more abundant in the inner leaflet in the plasma membrane, synthesized primarily in the mitochondria, and further metabolized in the endoplasmic reticulum. The decrease in PEs is rational due to the increase in oxidative stress, which will limit metabolic respiration and activity in the mitochondria. Decreases in plasmalogens, that is, vinyl ether PE species like PE(P‐16:0/18:1), are consistent with PE lipids being preferentially oxidized during conditions of oxidative stress. This dataset also shows that changes in lipid membrane domain composition and abundance may not be a universal change in the cell, more likely that key membrane regions and organelles that facilitate cell communication, transport, and metabolism may be impacted. These cellular processes are critical during development, which underscores how HIE can potentially result in lifelong injuries due to the lasting effects of poor neurodevelopment.

### 3.5 TGs Are Altered in Both DSM and DRM After HI

Figure 4E,F show an increase in TGs for the DRM, while the same species are decreased in the DSM. TGs play an important role for intracellular lipid droplet formation in order to facilitate protein trafficking and degradation. Accumulation of lipid droplets has been associated with inflammation and increased phagocytosis of cellular debris as a result of injury [48, 49]. This inhibits cells from maintaining their function, and as a result, can lead to apoptosis. TGs are associated with DRM for cellular organelles, such as the Golgi, which is responsible for either trafficking proteins to the plasma membrane or to lysosomes for degradation.^19^ Because of the dynamic nature of vesicle and lipid droplet formation, increasing the rigidity by the accumulation of TGs may prevent the regulation of these processes.

### 3.6 Lipids Solely Detected in the DRM Are Decreased After HI

**FIGURE 5:**
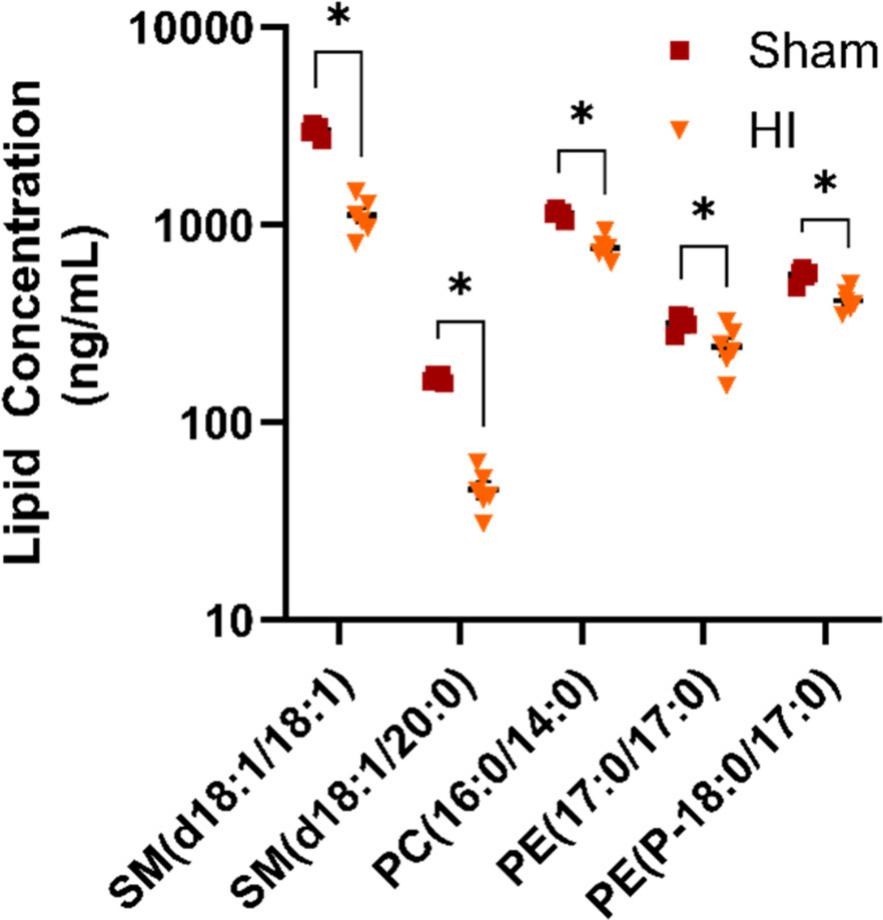
Lipid species only detectable in DRM changed after HI. Comparison between Sham and HI for lipid species only detected in DRM fraction that showed significant change based on injury. *p < 0.05 when compared using Student's *T*‐test.

In Figure 5, we show identified short‐chain (carbon number < 35) saturated glycerophospholipids and sphingomyelin that were only detected in DRM samples. This is to be expected as species are hypothesized to help maintain the rigidity of the membrane to limit permeability. All five lipids shown in Figure 5 are reduced after HIE, indicating that these lipids may be important during the disruption of the DRM after HIE. There are implications from this finding consistent with the disruption of neuronal development. Sphingomyelin is an important lipid for the myelination of axons in order to promote neuronal growth and development. This lipid class is most abundant in the brain and has been studied for its role in both cognitive impairment and motor function. Multiple studies have shown that disruption to sphingomyelin metabolism can result in neuronal cell death as well as the production of inflammatory cytokines from the microglia, and increase prostaglandin E2 (PGE2), which will contribute to neuroinflammation. Our findings are supported by literature detailing altered sphingolipid metabolism in other cognitive diseases [48, 50, 51].

### 3.7 Future Directions and Opportunities for Membrane Lipid Domain Analysis

Future studies might include modifications to the sample isolation, multi‐omic integration, investigation of subcellular organelle fractionation, and characterization of injury interventions. For example, since the isolation of the DRM and DSM domains was performed in this study, another group has published a method for the isolation of membrane domains using mass spectrometer‐friendly solvents [52]. Triton‐X 100 can interfere with MS analysis, and we are able to see residual detergent in samples that were analyzed. Poorly ionizable lipids like cholesterol esters, which would be expected in the DRM, are likely impacted by the sample preparation of the membrane domains. The combination of both proteomic and lipidomic data would enhance the comprehensive understanding of how lipid rafts may contribute to the different protein associations in the DRM and DSM domains. Proteomics data have revealed that proteins in the DRM have the potential to undergo palmitoylation at cysteine residues [51, 53]. Lipids can contribute the substrate for palmitoylation of DRM proteins based on co‐localization [54, 55, 56]. This would include the impact of palmitoylation of membrane proteins essential for signal transduction [57, 58]. Subcellular fractionation to study specific organelles may highlight differences that exist within the cell due to intracellular stress after hypoxia‐ischemia. Lastly, future directions may include how intervention strategies impact DRM lipid composition to better understand its role in the mechanism of HIE injury.

## 4 Conclusion

Lipid membrane domains are a complex and dynamic component of cell signaling. Traditional approaches of fluorescent microscopy have helped to characterize the size and timeframe that rigid domains exist. In vitro methods for studying lipid domains are not necessarily translatable due to the compositional abundance and diversity of endogenous lipids. Here, we sought to examine if advances in mass spectrometric analysis can lead to new hypotheses with lipid membrane domains for therapeutic intervention. Our findings are consistent with previous literature that has examined a number of different neurodegenerative diseases. We observed more differences with DRM fractions, which play an important role in neuronal growth and development. Lipid membrane domains are essential for cell signaling and protein trafficking. This study contributes to the understanding of membrane‐related disease mechanisms and potential therapeutic targets.

## Funding

This study was supported by the NIH/NICHD (P01 HD085928, PI–CFB) and by the University of Maryland School of Pharmacy Mass Spectrometry Center (SOP1841‐IQB2014, PI–MAK).

## Conflicts of Interest

The authors declare no conflicts of interest.

## Supporting information


**Table S1:** EquiSplash internal standard mass and retention time.
**Table S2:** Lipid identification parameters and data.
**Table S3:** Lipid class concentration and significance for DRM samples.
**Table S4:** Lipid class concentration and significance for DSM samples.
**Figure S1:** EquiSplash internal standard chromatogram.
**Figure S2:** Example PCA plot including QCs.
**Figure S3:** Heatmap of lipid changes in DRM after HI.
**Figure S4:** Heatmap of lipid changes in DSM after HI.

## Data Availability

The data that support the findings of this study are available from the corresponding author upon reasonable request.
