# Supplementary material for: Lipidomic Comparison of Detergent‐Soluble and Detergent‐Resistant Lipid Domains Isolated From Rat Cerebellum and the Effect of Hypoxia–Ischemia Using UPLC‐HDMSE
Source: J Mass Spectrom. 2026 Jan 5;61(1):e70000. doi: 10.1002/jms.70000 (PMC12770806; doi:10.1002/jms.70000)
Supplement: Supplementary file 1 — Table S1: EquiSplash internal standard mass and retention time. Table S2: Lipid identification parameters and data. Table S3: Lipid class concentration and significance for DRM samples. Table S4: Lipid class concentration and significance for DSM samples. Figure S1: EquiSplash internal standard chromatogram. Figure S2: Example PCA plot including QCs. Figure S3: Heatmap of lipid changes in DRM after HI. Figure S4: Heatmap of lipid changes in DSM after HI. [file JMS-61-e70000-s001.pdf]

Supporting Information

Lipidomic comparison of detergent-resistant and detergent-soluble lipid domains isolated from rat cerebellum and the effect of hypoxia-ischemia using UPLC-HDMS^{E}

Samuel A. Krug[1], Min He[2], Ningfeng Tang[2], Cynthia Bearer[2,3], Maureen A. Kane[1]*

[1] Department of Pharmaceutical Sciences, University of Maryland School of Pharmacy, Baltimore, MD 21201

[2] Department of Pediatrics, University of Maryland School of Medicine, Baltimore, MD 21201

[3] Current address: Department of Pediatrics, Case Western Reserve University School of Medicine, Cleveland, OH 44106

*Corresponding Author
Mailing Address: 20 N. Pine St, Rm N731, Baltimore, MD 21201
Email: mkane@rx.umaryland.edu
Telephone: 410-706-5097
Fax: 410-706-0886

This project was funded by NIH/NICHD P01 HD085928 (PI – CFB) and by the University of Maryland School of Pharmacy Mass Spectrometry Center (SOP1841-IQB2014, PI - MAK).

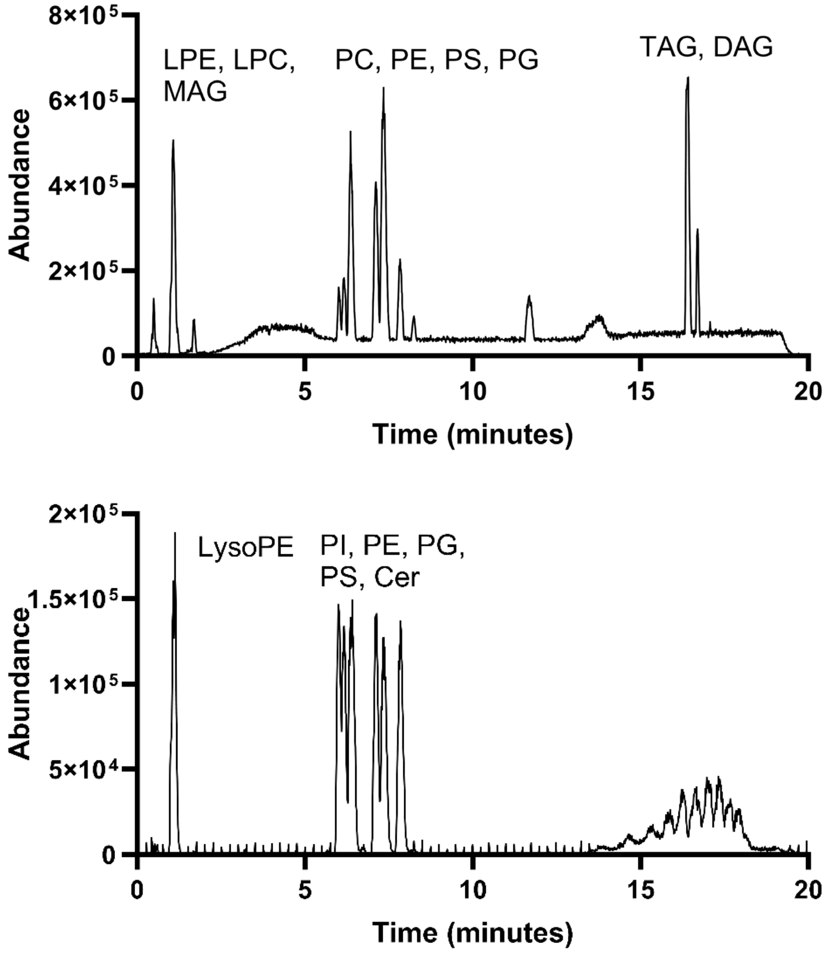

Supplemental Figure 1. **Example Chromatograms of EquiSplash Internal Standard supplemented in each sample**. EquiSplash Components are at a final concentration of 1 µg/mL. Consistency of retention time with known class standards were considered when assigning putative identification of lipid species. *Top* - Example extracted standard mix analyzed in ESI+ mode. *Bottom* – Example extracted standard mix analyzed in ESI- mode.

Supplemental Table 1 – List of Standards, exact mass, adduct, and retention time used to putatively assign lipid identification in Supplemental Table 1

			Neg		Pos					
Catalog Number	Mixture Component	Exact Mass(n)	M-H (rt)	M-H (mass)	M+H(rt)	M+H(mass)	M+NH4(rt)	M+NH4(mass)	M+Na(rt)	M+Na(mass)
791637	15:0-18:1(d7) PC	752.6061			**10.8**	753.6134				
791643	18:1(d7) Lyso PC	528.3921			**1.5**	529.3994				
791638	15:0-18:1(d7) PE	710.5591	**11.7**	709.5519	**11.7**	711.576				
791644	18:1(d7) Lyso PE	486.3451	**1.55**	485.3379	**1.6**	487.3683			1.6	509.34
791641	15:0-18:1(d7) PI (NH4 Salt)	829.5698	**7.6**	828.56					**7.66**	852.56
791639	15:0-18:1(d7) PS (Na Salt)	754.549	**8**	753.5417	**8.1**	755.5562			8.1	777.56
791648	15:0-18:1(d7)-15:0 TAG	811.7646					**17.5**	829.7985		
791649	d18:1-18:1(d9) SM	737.6397			**9.25**	738.647			9.25	760.62
860681	C15 Ceramide-d7	530.5404	**10.6**	529.5331					**10.6**	553.54

Supplemental Table 2: Complete List of All Identified Features that showed a p≤0.05 when comparing soluble or insoluble lipid fraction with Sham or HI Model. Lipids were putatively identified based on retention time in comparison with known class standards added to each sample, exact mass, and fragmentation patterns consistent with library entries.

Retention Time (min)	Mass *(m/z)*	Ionization Mode	Chemical Formula	Mass Error (ppm)	Drift Time (ms)	IDs	Adduct	Significant MS Peaks (decreasing sensitivity)	Database
0.85	500.2777	ESI-	C25H44NO7P	-1.15	3.96	PE(20:4/0:0)	M - H	303.2317 196.0354, 214.0458, 259.2401	LipidBlast
0.87	476.2762	ESI-	C27H46NO7P	-1.03	4.18	PE(18:2/0:0)	M-H	485.2654, 185.1874, 279.2296, 504.3083	LipidBlast
1.01	544.3365	ESI +	C26H52NO7P	-1.61	5.32	PC(P-16:0/2:0)	M + Na	496.3396 478.3275, 313.2719, 339.2870, 459.2463, 485.2619	LipidBlast
1.03	518.321	ESI +	C24H50NO7P	-1.48	5.26	PC(16:0/0:0)	M + Na	496.3398, 478.3277, 313.2719, 459.2458	LipidBlast, Metabolic Profiling CCS Library, NIST MS/MS Library
1.03	522.3549	ESI +	C26H52NO7P	-1.02	5.21	PC(18:1/0:0)	M + H	184.0747 511.3884, 166.0605, 496.3398, 492.3070	LipidBlast, Metabolic Profiling CCS Library, NIST MS/MS Library
1.04	506.3243	ESI -	C25H50NO7P	-1.86	3.91	PC(17:1/0:0)	M - H	331.2624, 225.0678	NIST MS/MS Library

**Retention Time (min)**	**Mass *(m/z)***	**Ionization Mode**	**Chemical Formula**	**Mass Error (ppm)**	**Drift Time (ms)**	**IDs**	**Adduct**	**Significant MS Peaks (decreasing sensitivity)**	**Database**
1.08	478.293	ESI -	C23H46NO7P	-1.82	4.18	PE(18:1/0:0)	M - H	289.2942 281.2466, 196.0354, 214.0458	LipidBlast, NIST MS/MS Library
1.49	524.3705	ESI +	C26H54NO7P	-1.09	5.37	PC(18:0/0:0)	M + H	184.0712 303.1570, 487.2772, 506.3566	LipidBlast, NIST MS/MS Library
1.57	480.3089	ESI -	C23H48NO7P	-1.29	4.34	PE(18:0/0:0)	M - H	283.2624 196.0347	LipidBlast
1.57	482.3235	ESI +	C23H48NO7P	-1.20	4.56	PE(18:0/0:0)	M + H	341.3061 403.2289, 310.3062, 166.0940	LipidBlast, NIST MS/MS Library
6.28	728.5209	ESI +	C38H76NO8P	1.15	7.97	GPCho(16:0/14:0)	M + Na	184.0749, 577.5629, 701.5565, 706.5406	NIST MS/MS Library
6.64	836.5453	ESI +	C46H78NO10P	2.03	8.57	GPSer(22:6/18:0)	M + H	651.5353 365.1026, 808.5862, 341.3017	LipidBlast, NIST MS/MS Library
6.67	835.5380	ESI -	C46H78NO10P	1.99	8.25	GPSer(18:0/22:6)	M-H	747.4981, 283.2619, 419.2554, 327.2303, 463.2221	LipidBlast, NIST MS/MS Library
6.78	758.5709	ESI +	C42H80NO89P	1.92	8.14	GPCho(20:1/14:1)	M + H	721.4761, 597.4831	LipidBlast, NIST MS/MS Library

Retention Time (min)	Mass (*m/z*)	Ionization Mode	Chemical Formula	Mass Error (ppm)	Drift Time (ms)	IDs	Adduct	Significant MS Peaks (decreasing sensitivity)	Database
6.89	740.5234	ESI +	C41H74NO8P	1.09	7.65	GPEtn(18:4/18:0)	M-H	365.1028, 599.5033, 313.2712	LipidBlast, NIST MS/MS Library
6.95	738.5087	ESI -	C41H74NO8P	1.09	7.27	GPEtn (16:0/20:4)	M - H	303.2311, 255.2303, 452.2747	LipidBlast, NIST MS/MS Library
7.04	885.5516	ESI -	C47H83O13P	2.00	8.95	GPIns(20:4/18:0)	M - H	303.2312, 419.2544, 283.2614, 241.0089, 581.3087	LipidBlast, NIST MS/MS Library
7.08	762.5276	ESI +	C40H76NO10P	-0.46	8.14	GPSer(16:0/18:1)	M + H	577.5184, 748.5293, 607.5078, 279.2656, 387.2772	LipidBlast, NIST MS/MS Library
7.12	748.5292	ESI +	C43H74NO7P	2.19	7.81	PE(P-16:0/22:6)	M + H	385.2722, 364.2591, 732.5539, 266.2814, 279.2652	LipidBlast, NIST MS/MS Library
7.19	746.5143	ESI -	C43H74NO7P	1.68	7.60	PE(16:0/22:6)	M - H	327.2314 283.2503, 436.2813, 255.2297, 418.2692	LipidBlast

Retention Time (min)	Mass *(m/z)*	Ionization Mode	Chemical Formula	Mass Error (ppm)	Drift Time (ms)	IDs	Adduct	Significant MS Peaks (decreasing sensitivity)	Database
7.22	810.5307	ESI -	C44H78NO10P	2.02	8.30	GPSer(20:4/18:0)	M - H	723.4966 283.2613, 419.2548 303.2299, 437.2650, 255.2291, 152.9928	LipidBlast, NIST MS/MS Library
7.22	830.5674	ESI +	C46H82NO8P	0.51	9.17	GPCho(20:5/18:0)	M + Na	808.5853, 784.5854, 653.5497	Metabolic Profiling CCS Library, LipidBlast
7.27	772.5295	ESI -	C45H78NO8P	1.04	7.76	GPEtn(18:0/22:6)	M - H2O - H	281.2451, 462.2975, 417.2374, 699.4960, 704.5222	LipidBlast, NIST MS/MS Library
7.52	748.5289	ESI -	C43H78NO8P	0.35	7.49	GPEtn(18:4/20:0)	M - H2O - H	327.2303, 283.2466, 438.2938	LipidBlast, NIST MS/MS Library
7.68	766.5393	ESI +	C43H76NO8P	1.59	7.97	GPEtn(18:1/20:4)	M + H	184.0713 753.6143, 625.5193	LipidBlast
7.73	764.5241	ESI -	C43H76NO8P	0.74	7.60	GPEtn(16:0/22:5)	M - H	329.2458, 255.2292, 285.2553	LipidBlast
7.82	722.5139	ESI -	C41H74NO7P	1.28	7.32	PE (P-16:0/20:4)	M - H	303.2311, 436.2802	LipidBlast

Retention Time (min)	Mass (*m/z*)	Ionization Mode	Chemical Formula	Mass Error (ppm)	Drift Time (ms)	IDs	Adduct	Significant MS Peaks (decreasing sensitivity)	Database
7.85	834.6018	ESI +	C48H84NO8P	1.25	8.95	GPCho(18:0/20:6)	M + H	184.0718 810.6022	NIST MS/MS Library
8.00	838.5608	ESI +	C46H80NO10P	1.83	8.68	GPSer(22:5/18:0)	M + H	653.5500, 629.5475, 784.5876	LipidBlast
8.05	836.5460	ESI -	C46H80NO10P	1.54	8.57	GPSer(18:0/22:5)	M - H	749.5129 283.2613, 419.2544, 437.2635, 329.2451, 152.9928	LipidBlast
8.28	734.5703	ESI +	C40H80NO8P	1.11	8.30	GPCho(16:0/16:0)	M + H	184.0757, 697.4787, 573.4851, 551.5028, 651.5349	LipidBlast, NIST MS/MS Library
8.45	790.5411	ESI -	C45H78NO8P	1.53	7.92	GPEtn(18:0/22:6)	M-H	327.2314, 283.2565, 480.3071	LipidBlast, NIST MS/MS Library
8.47	766.5396	ESI -	C43H78NO8P	0.45	7.60	GPEtn(18:0/20:4)	M-H	327.2313, 744.5560 283.2567, 480.3067, 331.2614	LipidBlast, NIST MS/MS Library
8.70	840.5762	ESI +	C46H82NO10P	1.51	8.79	GPSer(20:2/20:2)	M + H	655.5662	LipidBlast

Retention Time (min)	Mass (*m/z*)	Ionization Mode	Chemical Formula	Mass Error (ppm)	Drift Time (ms)	IDs	Adduct	Significant MS Peaks (decreasing sensitivity)	Database
8.72	748.5292	ESI -	C43H76NO7P	0.67	7.54	PE(P-16:0/22:5)	M - H	329.2460, 436.2800, 303.2281, 285.2560, 282.2499	LipidBlast
8.76	838.5618	ESI -	C46H82NO10P	1.70	8.63	GPSer(18:0/22:4)	M - H	751.5286, 283.2614, 419.2549, 331.2605, 437.2648, 152.9926	LipidBlast
9.04	718.5389	ESI +	C39H76NO8P	1.11	7.60	GPEtn(16:0/18:1)	M + H	627.5352, 577.5189	LipidBlast, NIST MS/MS Library
9.17	766.5401	ESI -	C43H78NO8P	1.16	7.65	GPEtn(20:4/18:0)	M - H	303.2315, 283.2623, 480.3074, 259.2397	LipidBlast, NIST MS/MS Library
9.38	788.5455	ESI -	C42H80NO10P	0.98	8.14	GPSer(18:1/18:0)	M-H	701.5121, 419.2536, 283.2599, 152.9927, 437.2640	LipidBlast, NIST MS/MS Library
9.39	718.5725	ESI +	C40H80NO7P	-2.76	8.08	PE(P-18:0/17:0)	M+H	385.2722, 392.2902, 635.5394, 307.2965, 294.3121	LipidBlast

Retention Time (min)	Mass (*m/z*)	Ionization Mode	Chemical Formula	Mass Error (ppm)	Drift Time	IDs	Adduct	Significant MS Peaks (decreasing sensitivity)	Database
9.40	776.5604	ESI +	C45H76NO7P	-0.35	8.14	PE(P-18:0/22:6)	M + H	385.2721 392.2902, 635.5393, 307.2968, 294.3123	LipidBlast, NIST MS/MS Library
9.47	752.5564	ESI +	C43H78NO7P	1.15	7.79	PE(P-16:0/22:4)	M + H	184.0711 389.3032, 364.2582, 611.5386	LipidBlast, NIST MS/MS Library
9.49	774.5456	ESI -	C45H78NO8P	1.69	8.12	PE(P-18:0/22:6)	M - H	327.2314, 464.3130, 446.3006, 284.2442	LipidBlast
9.57	750.5453	ESI -	C43H78NO7P	1.30	7.78	PE(P-16:0/22:4)	M-H	331.2624, 436.2809	LipidBlast
9.95	754.5747	ESI +	C43H80NO7P	0.29	8.03	PE (18:0/20:3)	M + H	613.5545 639.5689	LipidBlast
10.15	794.5706	ESI +	C45H80NO8P	1.45	8.35	GPEtn(20:2/20:3)	M + H	653.5505	LipidBlast
10.21	792.5557	ESI -	C43H78NO7P	1.02	7.97	GPEtn(22:5/18:0)	M - H	329.246, 283.261	LipidBlast
10.25	752.56	ESI +	C43H78NO7P	1.52	7.97	PE(P-18:0/20:4)	M + H	361.2718, 365.1019, 339.2867, 392.2894, 629.5485, 611.5376, 313.2712	LipidBlast, NIST MS/MS Library

**Retention Time (min)**	**Mass *(m/z)***	**Ionization Mode**	**Chemical Formula**	**Mass Error (ppm)**	**Drift Time**	**IDs**	**Adduct**	**Significant MS Peaks (decreasing sensitivity)**	**Database**
10.28	702.543	ESI +	C39H76NO7P	-0.26	7.60	PE(P-16:0/18:1)	M + H	361.2714 339.2867, 392.2897, 313.2706, 607.3890, 611.5378, 663.4518, 629.5502	Lipid Blast
10.29	778.5745	ESI +	C45H80NO7P	-0.04	8.30	PE(P-18:0/22:5)	M + H	361.2718, 392.2893, 752.5577, 611.5369, 387.2851, 415.3165	LipidBlast
10.35	750.5454	ESI -	C43H78NO7P	1.43	7.60	PE(P-18:0/20:4)	M-H	303.2311, 464.3125, 446.2998	LipidBlast
11.05	796.5864	ESI +	C45H82NO8P	-0.61	8.41	GPEtn(20:2/20:2)	M + H	655.5666, 358.3081	LipidBlast, Metabolic Profiling CCS Library
11.42	778.5746	ESI +	C45H80NO7P	0.11	8.35	PE(18:0/22:5)	M + H	387.2863, 392.2885	Lipid Blast
11.16	794.5716	ESI -	C45H82NO8P	1.32	8.03	GPEtn(22:4/18:0)	M - H	331.2621, 283.2613	LipidBlast, NIST MS/MS Library
11.49	776.5598	ESI -	C45H80NO7P	-0.15	7.92	PE(P-18:0/22:5)	M-H	329.2459, 464.3091	LipidBlast

Retention Time (min)	Mass (*m/z*)	Ionization Mode	Chemical Formula	Mass Error (ppm)	Drift Time	IDs	Adduct	Significant MS Peaks (decreasing sensitivity)	Database
7.22	720.5537	ESI +	C39H78NO8P	-0.09	7.92	GPEtn(17:0/17:0)	M + H	579.5336, 663.4517	LipidBlast
12.46	780.5888	ESI +	C45H82NO7P	-0.19	8.41	PE(18:0/22:4)	M + H	389.3027, 392.2893, 355.0665, 371.1008, 639.5696	LipidBlast
12.57	778.5764	ESI -	C45H82NO7P	0.97	7.97	PE(P-18:0/22:4)	M-H	331.2624, 464.3133	LipidBlast
13.83	808.6231	ESI +	C47H86NO7P	2.01	9.28	PE(P-18:0/24:4)	M + H	417.3335 264.2662, 184.0703, 355.0678, 371.0993, 392.2905, 341.0158, 281.0481	LipidBlast
16.12	872.7709	ESI +	C55H98O6	0.81	9.49	TG(16:1/18:1/18:2)	M + NH4	575.5018, 599.5020, 601.5164, 547.4696, 573.4857	LipidBlast
16.74	850.7875	ESI +	C53H100O6	2.01	10.36	TG(16:0/16:0/18:1)	M + NH4	551.5023, 577.5189, 603.5350, 523.4711, 824.7712	LipidBlast, NIST MS/MS Library
17.03	878.8195	ESI +	C55H104O6	2.74	10.63	TG(16:0/18:0/18:1)	M + NH4	579.5346, 551.5032, 605.5501, 577.5186, 607.5649, 852.8030	LipidBlast

Supplemental Table 3: Summary of putative lipid IDs including lipid class for detergent resistant (DRM) samples with the average concentration and standard error of the mean for each species. (n = 6 for both groups). Bold indicates a q-value of less than or equal to 0.05.

Lipid Class	Carbon Number	Degrees of Unsaturation	Putative ID Based on Fragmentation and Retention Time	Average Sham Detergent Resistant ng/mL	Average HI Detergent Resistant ng/mL	q-value
Ceramide	39	1	CerP(39:1)	168±3	74±7	**<0.01**
Sphingolipid	36	2	SM(d18:1/18:1)	1300±88	2995±87	**<0.01**
Sphingolipid	38	1	SM(d18:1/20:0)	2549±170	168±3	**<0.01**
Phosphatidylcholine	30	0	GPCho(16:0/14:0)	1134±23	765±39	**<0.01**
Phosphatidylcholine	32	0	GPCho(16:0/16:0)	33865±439	23659±1218	**<0.01**
Phosphatidylcholine	38	4	GPCho(18:0/20:4)	43±2	42±3	0.64
Phosphatidylcholine	44	6	GPCho(18:0/26:6)	663±31	679±33	0.50
Phosphatidylcholine	34	2	GPCho(20:1/14:1)	1884±65	1707±83	0.13
Phosphatidylcholine	38	5	GPCho(20:5/18:0)	16±1	14±1	0.15
Phosphatidylethanolamine	34	1	GPEtn(16:0/18:1)	1622±21	1099±21	**<0.01**
Phosphatidylethanolamine	36	4	GPEtn(16:0/20:4)	560±41	488±25	0.14
Phosphatidylethanolamine	38	5	GPEtn(16:0/22:5)	219±13	173±9	**0.02**
Phosphatidylethanolamine	34	0	GPEtn(17:0/17:0)	319±12	241±25	**0.03**
Phosphatidylethanolamine	38	4	GPEtn(18:0/20:4)	4988±266	4136±215	**0.04**
Phosphatidylethanolamine	40	6	GPEtn(18:0/22:6)a	2220±135	2065±98	0.27
Phosphatidylethanolamine	40	6	GPEtn(18:0/22:6)b	195±22	240±27	0.20
Phosphatidylethanolamine	38	4	GPEtn(18:1/20:3)	357±44	418±33	0.24
Phosphatidylethanolamine	38	5	GPEtn(18:1/20:4)	410±11	322±12	**<0.01**
Phosphatidylethanolamine	36	4	GPEtn(18:4/18:0)	757±31	615±28	**0.01**
Phosphatidylethanolamine	38	4	GPEtn(18:4/20:0)	197±11	136±5	**<0.01**
Phosphatidylethanolamine	40	4	GPEtn(20:2/20:2)	1208±51	961±60	**0.02**
Phosphatidylethanolamine	40	5	GPEtn(20:2/20:3)	867±27	593±24	**<0.01**
Phosphatidylethanolamine	38	4	GPEtn(20:4/18:0)	230±17	255±23	0.31
Phosphatidylethanolamine	40	4	GPEtn(22:4/18:0)	776±44	731±55	0.39
Phosphatidylethanolamine	40	5	GPEtn(22:5/18:0)	509±25	392±21	**0.01**
Phosphatidylinositol	38	4	GPIns(20:4/18:0)	3255±273	3371±266	0.52
Phosphatidyserine	36	1	GPSer(18:0/18:1)	765±36	611±45	**0.03**
Phosphatidyserine	40	4	GPSer(18:0/22:4)	645±49	615±68	0.50
Phosphatidyserine	40	5	GPSer(18:0/22:5)	491±41	389±28	**0.06**

Supplemental Table 3 Continued.

Lipid Class	Carbon Number	Degrees of Unsaturation	Putative ID Based on Fragmentation and Retention Time	Average Sham Detergent Resistant ng/mL	Average HI Detergent Resistant ng/mL	q-value
Phosphatidyserine	40	6	GPSer(18:0/22:6)	2651±222	2408±128	0.26
Phosphatidyserine	40	4	GPSer(20:2/20:2)	913±52	754±62	0.09
Phosphatidyserine	38	4	GPSer(20:4/18:0)	563±49	499±42	0.26
Phosphatidyserine	40	5	GPSer(22:5/18:0)	694±36	474±31	**<0.01**
Phosphatidyserine	40	6	GPSer(22:6/18:0)	3369±155	2629±131	**<0.01**
Phosphatidyserine	34	1	GPSer(16:0/18:1)	317±8	214±11	**<0.01**
Lysophophatidylcholine	16	0	PC(16:0/0:0)	117±4	89±9	**0.03**
Lysophophatidylcholine	17	1	PC(17:1/0:0)	48±8	70±13	0.17
Lysophophatidylcholine	18	0	PC(18:0/0:0)	382±12	287±15	**<0.01**
Lysophophatidylcholine	18	1	PC(18:1/0:0)a	62±8	106±17	0.06
Lysophophatidylcholine	18	1	PC(18:1/0:0)c	683±57	953±104	0.06
Lysophophatidylcholine	20	4	PC(20:4/0:0)	34±5	73±10	**0.01**
Lysophophatidylethanolamine	18	0	PE(18:0/0:0)a	829±47	581±62	**0.02**
Lysophophatidylethanolamine	18	0	PE(18:0/0:0)b	821±41	468±33	**<0.01**
Lysophophatidylethanolamine	18	1	PE(18:1/0:0)	381±41	455±58	0.26
Lysophophatidylethanolamine	20	4	PE(20:4/0:0)	1314±160	1559±175	0.26
Phosphatidylethanolamine	34	1	PE(P-16:0/18:1)	605±8	441±14	**<0.01**
Phosphatidylethanolamine	36	4	PE(P-16:0/20:4)	643±44	578±46	0.26
Phosphatidylethanolamine	38	4	PE(P-16:0/22:4)a	565±36	506±45	0.26
Phosphatidylethanolamine	38	4	PE(P-16:0/22:4)b	780±34	616±39	**0.02**
Phosphatidylethanolamine	38	5	PE(P-16:0/22:5)	208±12	155±11	**0.01**
Phosphatidylethanolamine	38	6	PE(P-16:0/22:6)a	1479±101	1276±72	0.12
Phosphatidylethanolamine	38	6	PE(P-16:0/22:6)b	2614±93	2065±98	**<0.01**
Phosphatidylethanolamine	35	0	PE(P-18:0/17:0)	555±18	414±22	**<0.01**
Phosphatidylethanolamine	38	3	PE(P-18:0/20:3)	220±5	154±7	**<0.01**
Phosphatidylethanolamine	38	4	PE(P-18:0/20:4)a	990±56	869±58	0.15
Phosphatidylethanolamine	38	4	PE(P-18:0/20:4)b	1339±47	997±50	**<0.01**

Supplemental Table 3 continued.

Lipid Class	Carbon Number	Degrees of Unsaturation	Putative ID Based on Fragmentation and Retention Time	Average Sham Detergent Resistant ng/mL	Average HI Detergent Resistant ng/mL	q-value
Phosphatidylethanolamine	40	4	PE(P-18:0/22:4)a	412±27	350±28	0.14
Phosphatidylethanolamine	40	4	PE(P-18:0/22:4)b	603±25	424±22	**<0.01**
Phosphatidylethanolamine	40	5	PE(P-18:0/22:5)a	151±9	106±9	**0.01**
Phosphatidylethanolamine	40	5	PE(P-18:0/22:5)b	125±7	100±9	0.06
Phosphatidylethanolamine	40	5	PE(P-18:0/22:5)c	240±8	146±8	**<0.01**
Phosphatidylethanolamine	40	6	PE(P-18:0/22:6)a	1909±121	1682±106	0.16
Phosphatidylethanolamine	40	6	PE(P-18:0/22:6)b	2683±99	2115±99	**<0.01**
Phosphatidylethanolamine	42	4	PE(P-18:0/24:4)	108±3	60±3	**<0.01**
Triglyceride	50	1	TG(16:0/16:0/18:1)	578±20	479±12	**<0.01**
Triglyceride	52	1	TG(16:0/18:0/18:1)	218±9	186±5	**0.02**
Triglyceride	52	4	TG(16:1/18:1/18:2)	140±9	87±3	**<0.01**

Supplemental Table 4: Summary of putative lipid IDs including lipid class for detergent soluble (DSM) samples with the average concentration and standard error of the mean for each species. (n = 6 for HI, n=5 for Sham). Bold indicates a q-value of less than or equal to 0.05.

Lipid Class	Carbon Number	Degrees of Unsaturation	Putative ID Based on Fragmentation and Retention Time	Average Sham Detergent Soluble ng/mL	Average HI Detergent Soluble ng/mL	q-value
Phosphatidylcholine	32	0	GPCho(16:0/16:0)	5993±212	6404±125	**<0.01**
Phosphatidylcholine	44	6	GPCho(18:0/26:6)	1418±56	1659±47	0.72
Phosphatidylcholine	34	2	GPCho(20:1/14:1)	2460±84	2710±84	0.14
Phosphatidylethanolamine	34	1	GPEtn(16:0/18:1)	1065±46	1216±27	**<0.01**
Phosphatidylethanolamine	36	4	GPEtn(16:0/20:4)	847±23	1004±16	0.16
Phosphatidylethanolamine	38	5	GPEtn(16:0/22:5)	292±14	340±12	**0.01**
Phosphatidylethanolamine	38	4	GPEtn(18:0/20:4)	7213±186	8222±88	**0.03**
Phosphatidylethanolamine	40	6	GPEtn(18:0/22:6)a	3876±130	4437±68	0.37
Phosphatidylethanolamine	40	6	GPEtn(18:0/22:6)b	810±35	922±22	0.24
Phosphatidylethanolamine	38	4	GPEtn(18:1/20:3)	628±28	853±49	0.29
Phosphatidylethanolamine	38	5	GPEtn(18:1/20:4)	527±20	610±17	**<0.01**
Phosphatidylethanolamine	36	4	GPEtn(18:4/18:0)	1075±35	1238±34	**0.01**
Phosphatidylethanolamine	38	4	GPEtn(18:4/20:0)	375±15	420±13	**<0.01**
Phosphatidylethanolamine	40	4	GPEtn(20:2/20:2)	2014±93	2360±62	**0.01**
Phosphatidylethanolamine	40	5	GPEtn(20:2/20:3)	1336±73	1550±32	**<0.01**
Phosphatidylethanolamine	38	4	GPEtn(20:4/18:0)	388±13	455±8	0.43
Phosphatidylethanolamine	40	4	GPEtn(22:4/18:0)	1372±49	1722±64	0.55
Phosphatidylethanolamine	40	5	GPEtn(22:5/18:0)	824±30	997±31	**0.01**
Phosphatidylinositol	38	4	GPIns(20:4/18:0)	5478±153	6028±138	0.77
Phosphatidyserine	36	1	GPSer(18:0/18:1)	871±26	1044±15	**0.03**
Phosphatidyserine	40	4	GPSer(18:0/22:4)	1355±43	1523±25	0.74
Phosphatidyserine	40	5	GPSer(18:0/22:5)	951±35	1061±28	0.06
Phosphatidyserine	40	6	GPSer(18:0/22:6)	4461±110	5079±83	0.35
Phosphatidyserine	40	4	GPSer(20:2/20:2)	1721±66	1885±46	0.09
Phosphatidyserine	38	4	GPSer(20:4/18:0)	1187±36	1286±35	0.34
Phosphatidyserine	40	5	GPSer(22:5/18:0)	1196±51	1254±39	**<0.01**
Phosphatidyserine	40	6	GPSer(22:6/18:0)	5305±166	5856±181	**0.01**
Phosphatidyserine	34	1	GPSer(16:0/18:1)	360±13	410±12	**<0.01**
Lysophophatidylcholine	16	0	PC(16:0/0:0)	557±48	552±44	**0.02**
Lysophophatidylcholine	17	1	PC(17:1/0:0)	986±47	1027±29	0.20

Supplemental Table 4 Continued

Lipid Class	Carbon Number	Degrees of Unsaturation	Putative ID Based on Fragmentation and Retention Time	Average Sham Detergent Soluble ng/mL	Average HI Detergent Soluble ng/mL	q-value
Lysophophatidylcholine	18	0	PC(18:0/0:0)	1018±53	924±27	**<0.01**
Lysophophatidylcholine	18	1	PC(18:1/0:0)a	1561±72	1626±25	0.06
Lysophophatidylcholine	18	1	PC(18:1/0:0)b	304±25	318±26	0.12
Lysophophatidylcholine	18	1	PC(18:1/0:0)c	9313±323	9474±207	0.06
Lysophophatidylcholine	20	4	PC(20:4/0:0)	1129±44	1169±22	**0.01**
Phosphatidylcholine	18	0	PC(P-16:0/2:0)	605±24	661±19	**<0.01**
Lysophophatidylethanolamine	18	0	PE(18:0/0:0)a	3416±115	3037±91	**0.01**
Lysophophatidylethanolamine	18	0	PE(18:0/0:0)b	2813±118	2391±63	**<0.01**
Lysophophatidylethanolamine	18	1	PE(18:1/0:0)	5190±209	5077±109	0.34
Lysophophatidylethanolamine	20	4	PE(20:4/0:0)	16645±437	16605±301	0.34
Lysophophatidylethanolamine	22	5	PE(22:5/0:0)	2305±97	2499±76	**0.01**
Phosphatidylethanolamine	34	1	PE(P-16:0/18:1)	561±30	662±15	**<0.01**
Phosphatidylethanolamine	36	4	PE(P-16:0/20:4)	1516±67	1712±33	0.34
Phosphatidylethanolamine	38	4	PE(P-16:0/22:4)a	1313±44	1524±37	0.34
Phosphatidylethanolamine	38	4	PE(P-16:0/22:4)b	1673±91	1871±31	**0.01**
Phosphatidylethanolamine	38	5	PE(P-16:0/22:5)	495±19	571±22	**0.01**
Phosphatidylethanolamine	38	6	PE(P-16:0/22:6)a	3041±108	3486±62	0.13
Phosphatidylethanolamine	38	6	PE(P-16:0/22:6)b	5110±169	5801±139	**<0.01**
Phosphatidylethanolamine	38	3	PE(P-18:0/20:3)	365±20	416±9	**<0.01**
Phosphatidylethanolamine	38	4	PE(P-18:0/20:4)a	2218±68	2613±61	0.17
Phosphatidylethanolamine	38	4	PE(P-18:0/20:4)b	2752±161	3007±60	**<0.01**
Phosphatidylethanolamine	40	4	PE(P-18:0/22:4)a	975±37	1198±43	0.15
Phosphatidylethanolamine	40	4	PE(P-18:0/22:4)b	1348±62	1544±64	**<0.01**
Phosphatidylethanolamine	40	5	PE(P-18:0/22:5)a	348±16	422±19	**0.01**
Phosphatidylethanolamine	40	5	PE(P-18:0/22:5)b	346±24	378±12	0.06
Phosphatidylethanolamine	40	5	PE(P-18:0/22:5)c	510±24	605±21	**<0.01**
Phosphatidylethanolamine	40	6	PE(P-18:0/22:6)a	3987±138	4704±100	0.19
Phosphatidylethanolamine	40	6	PE(P-18:0/22:6)b	5368±235	6332±113	**<0.01**
Phosphatidylethanolamine	42	4	PE(P-18:0/24:4)	180±10	222±8	**<0.01**
Triglyceride	50	1	TG(16:0/16:0/18:1)	309±8	256±4	**<0.01**
Triglyceride	52	1	TG(16:0/18:0/18:1)	101±3	86±2	**0.01**
Triglyceride	52	4	TG(16:1/18:1/18:2)	81±4	75±4	**<0.01**

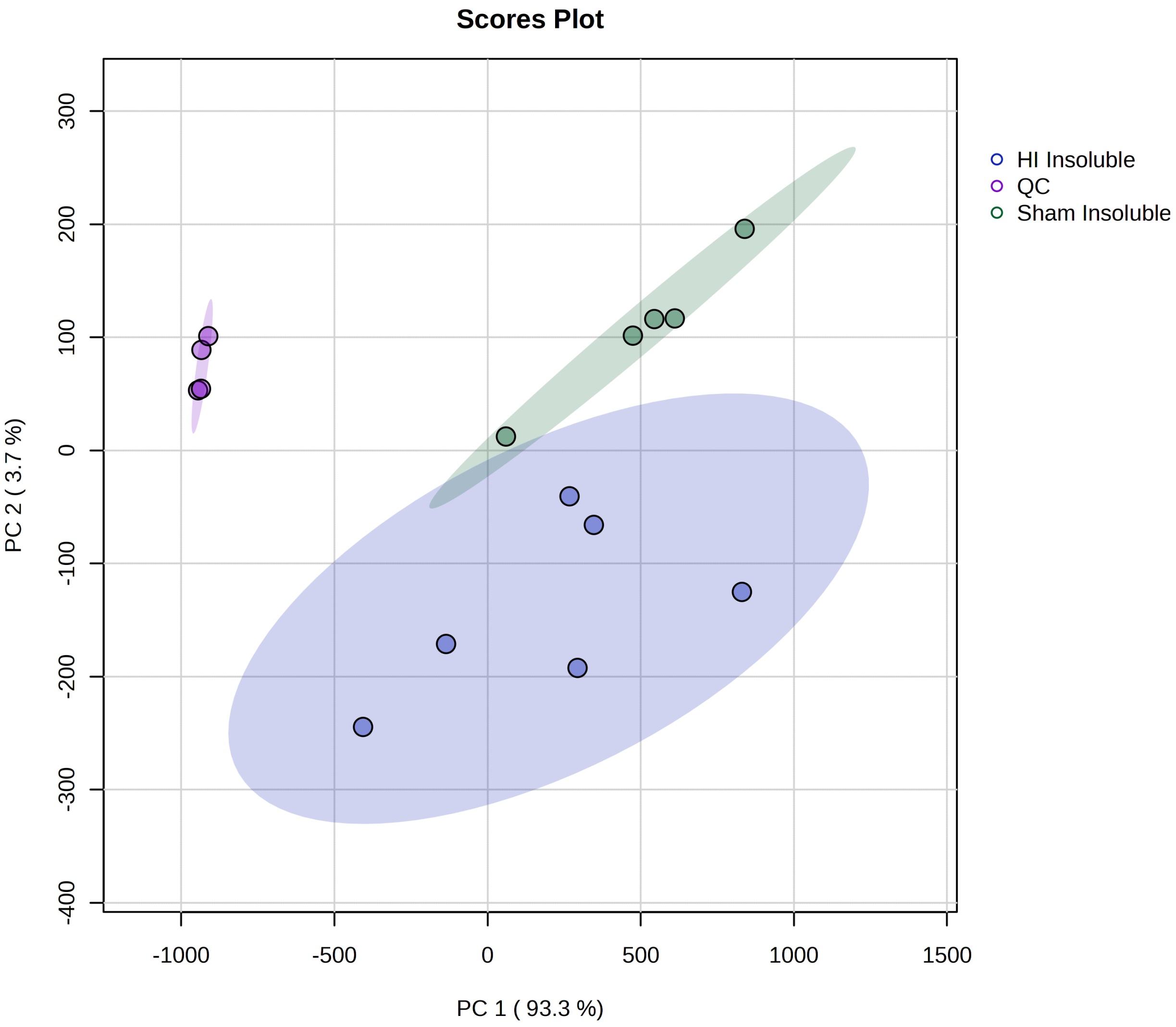

Supplemental Figure 2. PCA Plot Comparing HI Insoluble (DRM) (Blue), Sham Insoluble (DRM) (Green), and QCs (Purple). PCA displayed a total of 97.0% of variance attributed to PC1 (93.3%) and PC2 (3.7%) components. Elliptical patterns show the 95% confidence interval for each group.

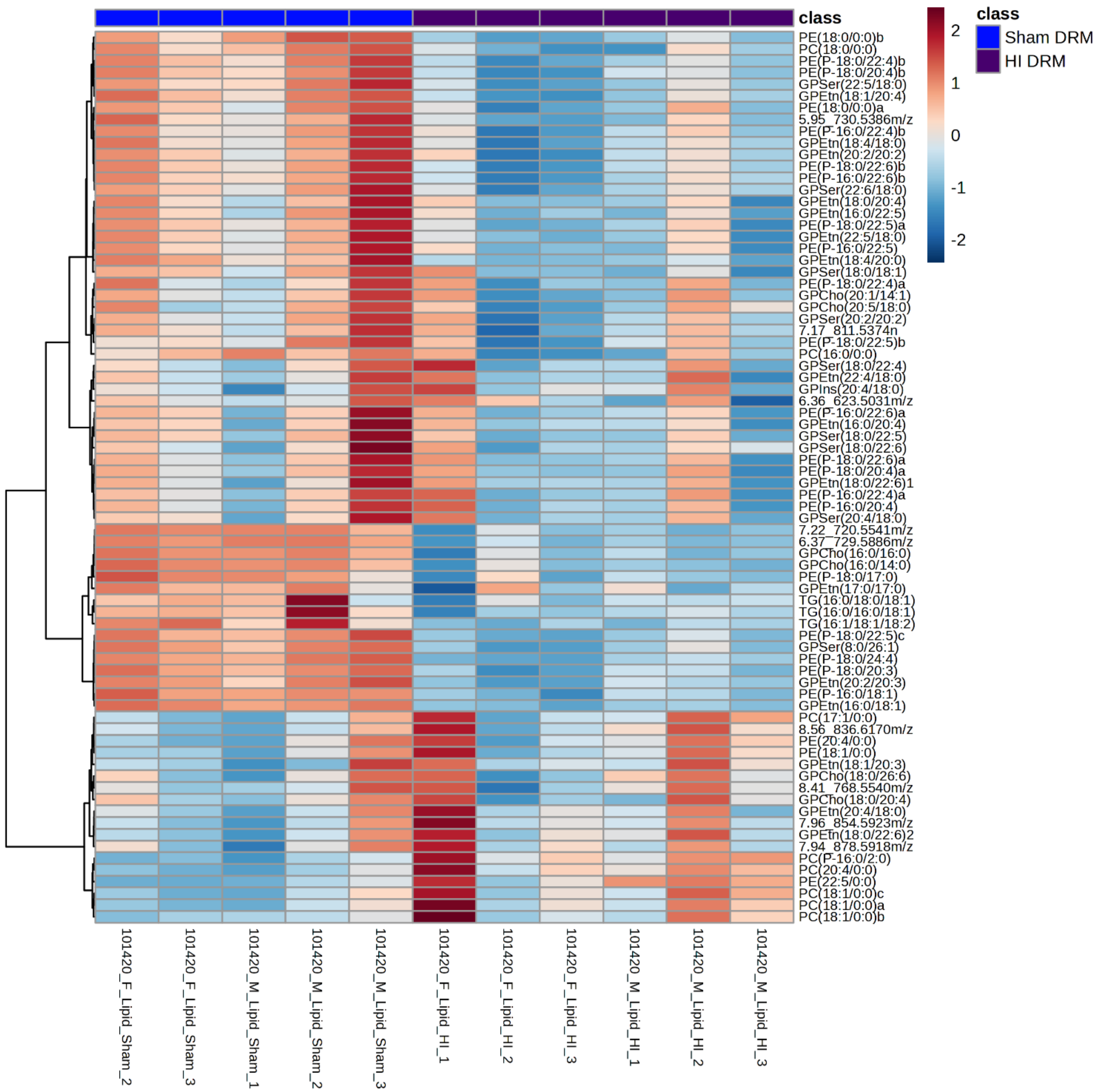

Supplemental Figure 3: **Heat map of lipid changes in DRM after HI.** Hierarchical (Euclidean Ward) Clustering of Sham (Blue) and HI (Purple) for all significantly different ($p < 0.05$) features in MetaboAnalyst 6.0.

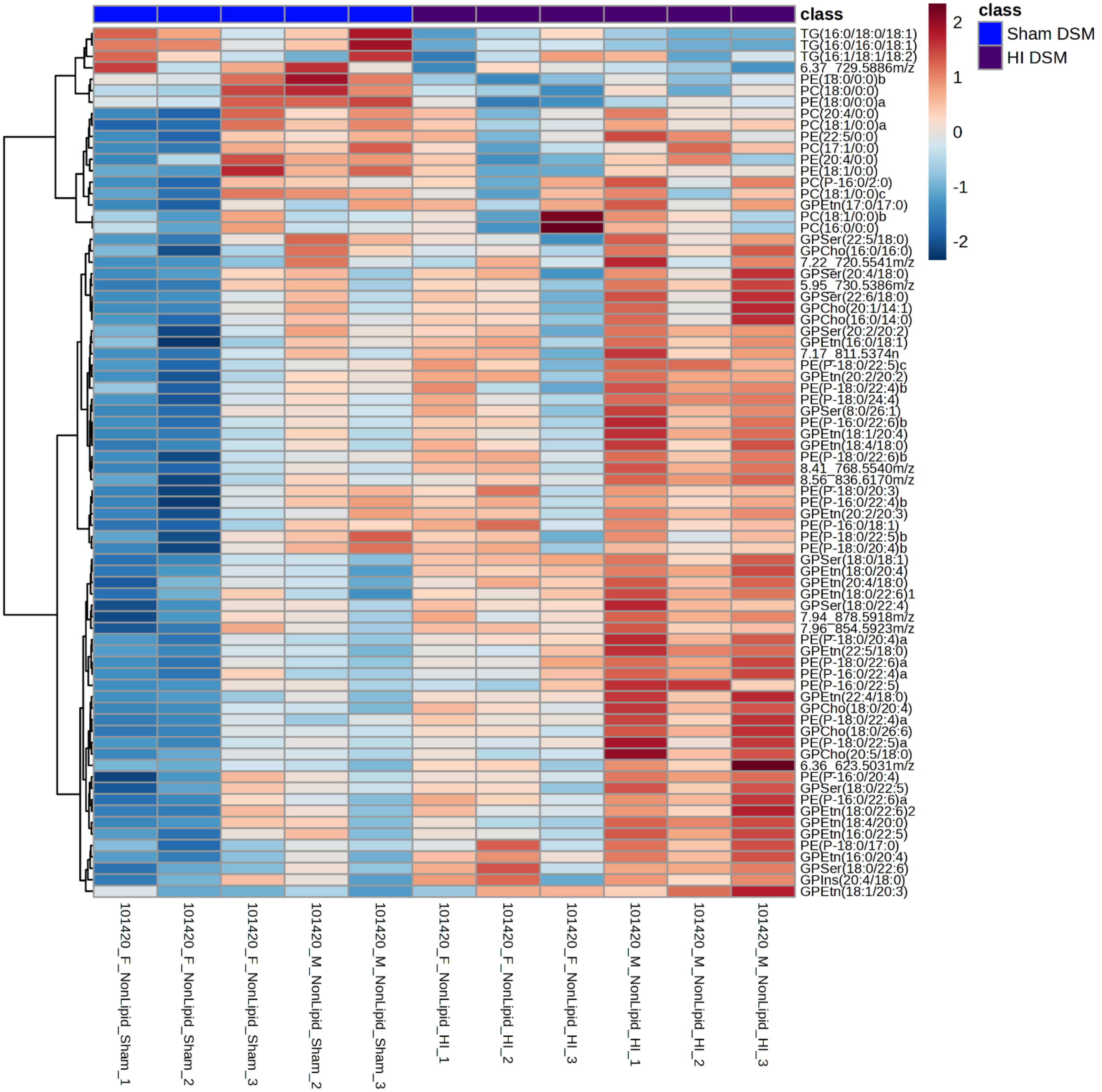

Supplemental Figure 4: **Heat map of lipid changes in DSM after HI.** Hierarchical (Euclidean Ward) Clustering of Sham (Blue) and HI (Purple) for all significantly different ($p < 0.05$) features in MetaboAnalyst 6.0.
